# Does the IOFix implant improve union rates?

**DOI:** 10.1186/s12891-020-03689-1

**Published:** 2020-10-06

**Authors:** David Segal, Nissim Ohana, Meir Nyska, Ezequiel Palmanovich

**Affiliations:** 1 Department of Orthopaedic Surgery, Meir Medical Center, 59 Tchernichovski st, Kfar Saba, Israel; 2 Tel Aviv University, Tel Aviv, Israel

**Keywords:** Hallux rigidus, IOFix, Union, Fusion

## Abstract

**Background:**

First metatarso-phalangeal joint fusion is the current gold standard for severe hallux rigidus. Data regarding the union rate and the re-operation rate when IOFix (an Intra-osseous fixation device, Extremity medical, New Jersey, USA) is used for hallux rigidus fusion is limited but promising. The aim of this study was to review our outcomes with the IOFix implant.

**Methods:**

We have conducted a retrospective chart review, following the approval of the hospital IRB committee. Exclusion criteria included bilateral operations on the same patient, multiple surgeries, charcot foot or other structural foot abnormalities (except hallux valgus), rheumatoid arthritis and a recent foot trauma. We collected demographic data, physical examination documentation, functional score evaluations (AOFAS), and Plain radiographic studies.

**Results:**

Thirty patients were included in the study. The mean age was 60.36 ± 9.12 (range 36 to 77) years, 18 (60%) female patients and 12 (40%) male. Fourteen (53.33%) were left side pathologies. The average follow up period was 36.2 ± 12.31 (range 12 to 54) months. Union was obtained in 28 (93.33%) patients, of whom none had requested a hardware removal due to a prominent hardware during a minimum of 2 year follow up period. The mean postoperative AOFAS score was 80.5 ± 10.87 (range 35 to 90). A more stringent inclusion criteria and fusion definitions would have led to an exclusion of two more patients and a dropout of two patients from the “fused” group, which would have led to a fusion rate of 85.71%.

**Conclusions:**

This is the largest series of hallux rigidus patients that were operated with an IOFix device. The rates of fusion and hardware removal in MTPJ1 arthrodesis performed with an IOFix implant were found to be similar at most when compared to previously described rates that were obtained with other cheaper and more simple fixation devices.

**Level of evidence:**

4

## Background

Hallux rigidus (HR) is a common foot pathology that is associated with degenerative changes of the first metatarso-phalangeal joint (MTPJ1) and causes a limitation in hallux dorsiflexion [1, 2]. To date the surgical options to treat HR include cheilectomy, excisional arthroplasty, interposition arthroplasty [3], phalangeal osteotomy, first metatarsal osteotomy, implant arthroplasty, and arthrodesis [4, 5]. MTPJ1 fusion, which has been described in various techniques, allows a definitive resolution and is the current gold standard for severe MTPJ1 osteoarthritis [1, 2, 5]. During arthrodesis the joint is prepared with flat or conical cuts, often in a ball and socket configuration which are performed with reamers in order to obtain optimal bone contact and stability. A few fixation devices were described in order to obtain additional stability in the fused joint: a lag screw, a dorsal plate, crossed screws, staples and an intraosseous device [6]. The union rate with various implants was found to be consistently around 91 to 100% [7–14] and the need for reoperation due to a prominent hardware was usually between 0 and 13% [6–14] (with the exception of 78% in one study [15]). These reports imply a relative success in obtaining union, but in some cases, as in a single screw implant or a dorsal plate, the secondary procedure rates seem to be too high.

The IOFIX (an Intra-osseous fixation device, Extremity medical, New Jersey, USA) is a fixed or variable angle intraosseous device that is consisted of an “X-post” that is inserted proximal and parallel to the joint, and a compression screw that passes through a hole in the post, crosses the joint and engages on the proximal phalanx. Data regarding the fusion rate and the re-operation rate when IOFIX is used is limited but promising [6]. In a recent biomechanical study [16] it was found that the intramedullary device demonstrated the highest initial compression force when compared to a plantar locking plate and a dorsal locking plate. Nevertheless, it was also found to be the most susceptible to failure. The authors concluded that further research with clinical data is necessary in order to further analyze the outcomes of this device. The aim of this study was to review the fusion rate of the IOFix and the removal rate following fusion due to prominent hardware based our experience.

## Methods

We have conducted a retrospective chart review, following the approval of our hospital IRB committee (the full name will be revealed for the unblinded version of the manuscript). We included patients aged 18 to 99 who were operated for a moderate to severe MTPJ1 osteoarthritis (OA) related HR [2] in a single medical center by a single surgeon (name initials will be added in the un-blinded manuscript) between the years 2015–2017 and who had pre-operative weight baring Plain radiographs. Exclusion criteria were bilateral operations on the same patient, multiple foot surgeries, charcot foot or other structural foot abnormalities (except hallux valgus), rheumatoid arthritis and a recent foot trauma. We collected demographic data, physical examination documentation, pre-operative and the latest postoperative (36.2 ± 12.31 (range 12 to 54) months) functional score evaluations (AOFAS) [17]. Plain radiographic studies were taken at the following postoperative time points: 6 weeks, 3–6-12 months, and at 24 months when applicable. The consecutive radiographs were used in order to evaluate the union rate [8]. When reoperations were conducted, the reasons that have led to these procedures, as well as the surgical records were studied.

**Fig. 1:**
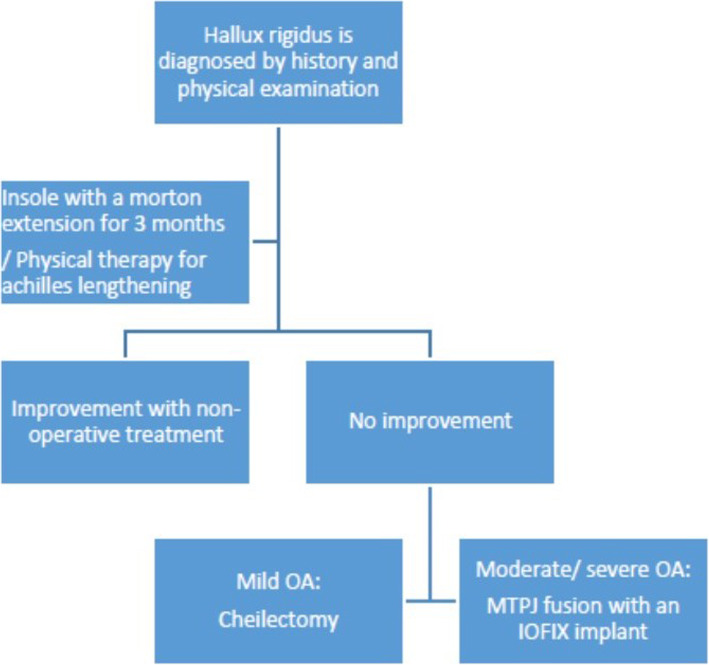
A treatment protocol for hallux rigidus

**Fig. 2:**
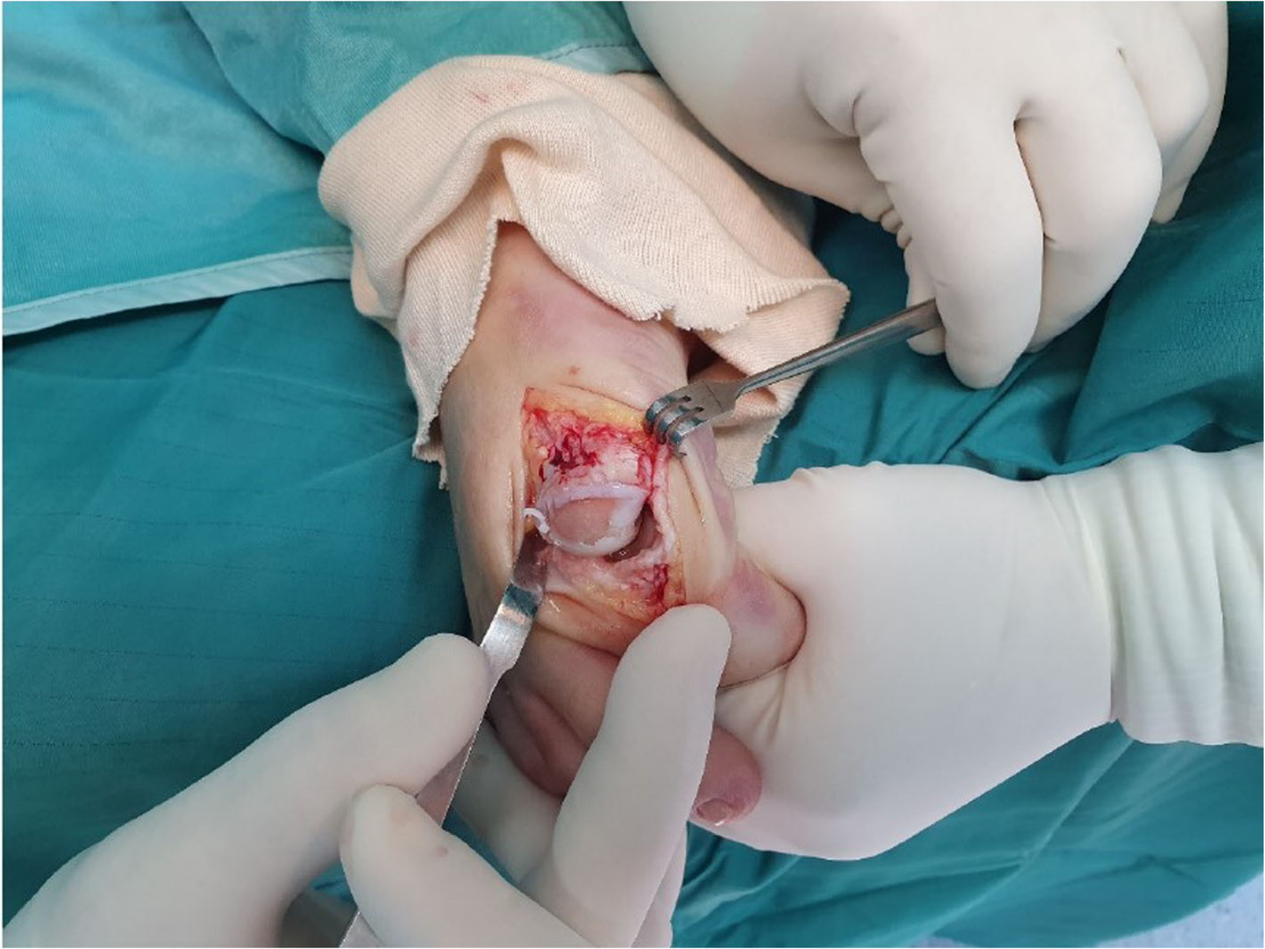
An intra-operative photo of a first metatarsophalangeal joint showing a destruction of more than 50% of the cartilage on the metatarsal side. This finding lead to a decision to fuse the joint rather than to conduct a cheilectomy procedure

**Fig. 3:**
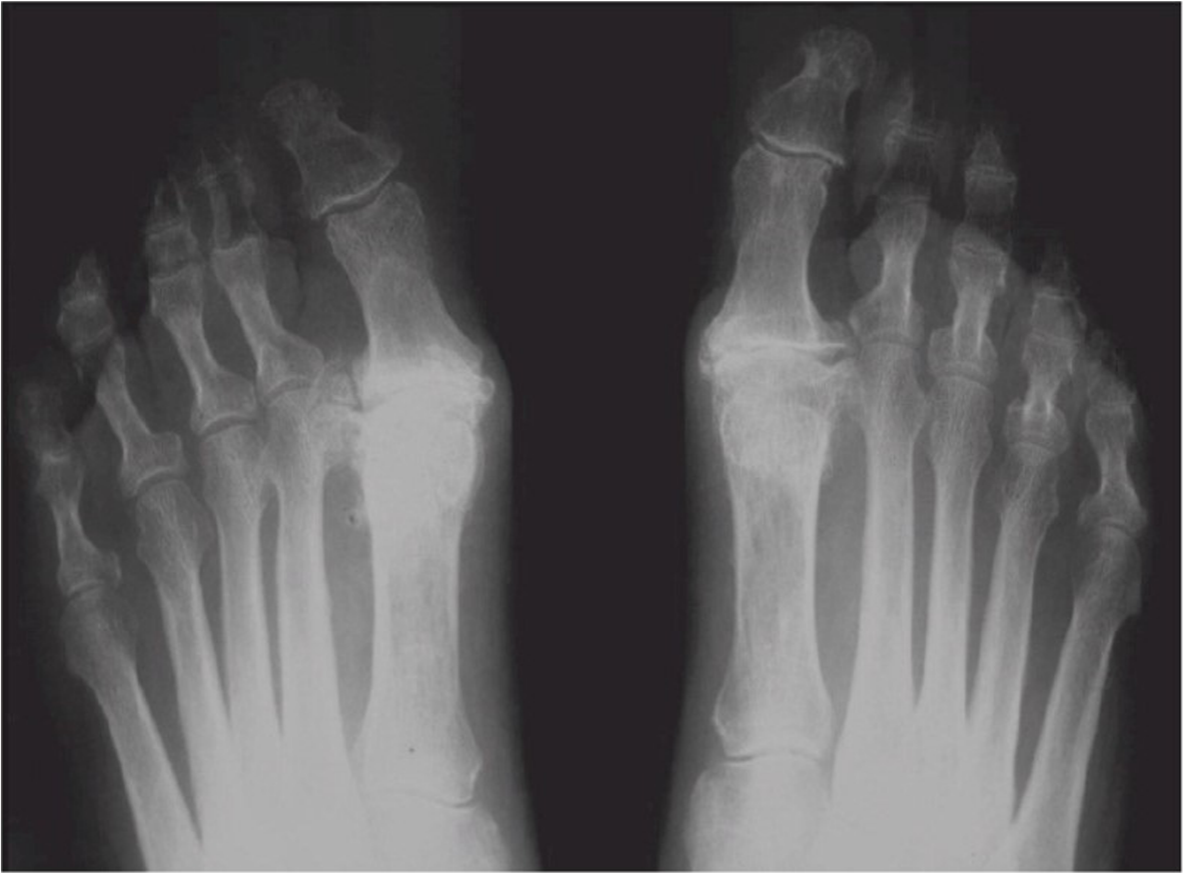
A standing X ray radiograph of a patient with bilateral hallux rigidus that was considered as severe

A flow chart that demonstrates our treatment protocol is presented in Fig. 1. Each patient was classified clinically and radiographically into one of 3 categories: mild, moderate or severe HR. [2] We did not offer surgical treatment to mild OA patients. When OA was considered to be moderate, surgical treatment was advised. In these cases we have accepted the patients’ informed consent for both a cheilectomy procedure and an MTPJ1 fusion [8]. The specific procedure was then chosen during the operation and was based on the remnant cartilage state as was evaluated intraoperatively. If sufficient cartilage was seen, which was estimated as > 50% of the joint surface, a cheilectomy was performed. If the remnant cartilage was found to be insufficient (Fig. 2), or if the HR grade was pre-operatively classifies as severe Fig. 3), an MTPJ1 fusion was performed. Only patients who have underwent MTPJ1 fusion and have answered our inclusion criteria were included in this study.

The MTPJ1 fusion was conducted with the patient in a supine position, under an ankle block anaesthesia and an above ankle tourniquet. An image intensifier was used throughout the procedure. A standard dorsal incision was used. After the joint was revealed we used conical reamers and stabilized the joint with 1.6 K-wires. At this point the IOFix was inserted using the standard surgical technique [18]. Following surgery we restricted weight bearing to the heels only, using a firmed sole shoe and crutches. Patients were invited for a follow up examinations on postoperative weeks 2, 4, 6, 12, 24 and 52.

Union was defined both clinically and radiographically, in concordance with previous publications [6]. Clinically, we expected the patients to be able to fully bear weight on their feet without pain, and to deny pain when applying external force on the 1st MTPJ. Radiographically we searched for a complete callus formation or trabeculae on 3 out of 4 cortices. If at 6 months follow up no union was seen on an Plain radiograph and there was a mild local discomfort, a 6 months therapy course with MELMAK™^,^ a low intensity pulsed ultrasound (LIPUS) device, (BTT Melmak Development & production GmbH. Raisting Germany) was indicated in order to encourage union.

## Results

**Fig. 4:**
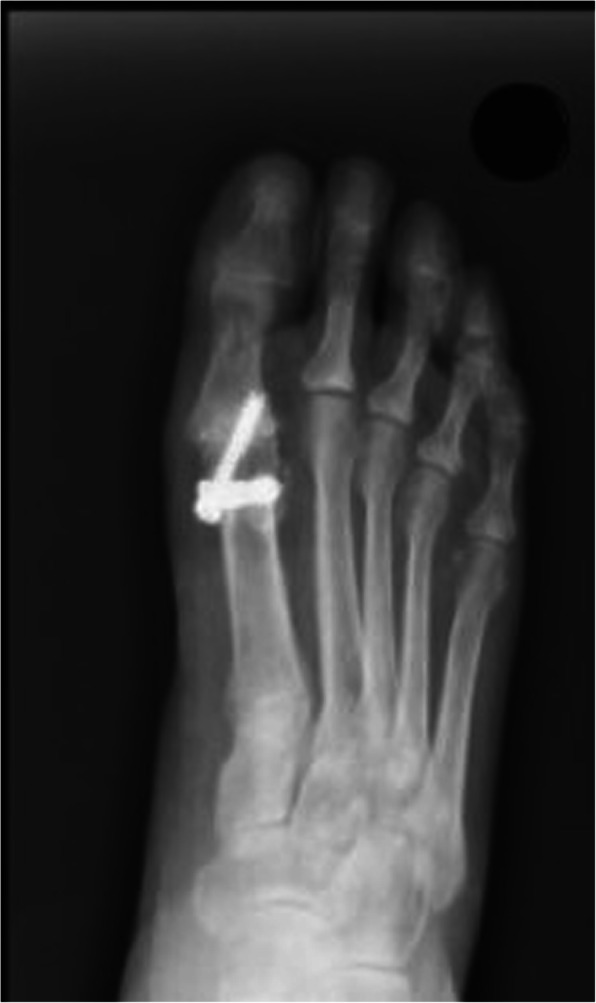
A standing X ray radiograph of a patients who was operated for a hallux rigidus with an IOFix implant. This patients suffered from a hardware protrusion. In this case a technique failure has led to the unwanted complication

Thirty patients were included in the study (Table 1). The mean age was 60.36 ± 9.12 (range 36 to 77) years, 18 (60%) female and 12 (40%) male. Fourteen (53.33%) had a left side pathology. The average follow up period was 36.2 ± 12.31 (range 12 to 54) months. Union was achieved in 28 (93.33%) patients, of whom none had requested a hardware removal due to a prominent hardware during a minimum of 2 year follow up period. The mean time to union was 8.46 ± 2.93 (range 6 to 20) weeks. The mean postoperative AOFAS scores were found to be 80.5 ± 10.87 (range 35 to 90) points. Due to delayed union signs, 2 patients were treated with a 6 months course of MELMAK™. Following this treatment union was obtained in both patients. Two patients reported a discomfort due to a MTPJ1 rigidity. In one patient the hardware could be palpated by the surgeon at a follow up visit, even though the patient could not feel it and was not symptomatic. One patient reported a mild pain that was regarded to a post protrusion (Fig. 4). Two patients were lost to follow up at 6 months, but we do know that at their last visit union was seen on an Plain radiograph and that at 2 years they did not undergo a repeat surgery at any of the national public health system hospitals or at the largest private medical care facility at our area.

**Table 1:** Patients who underwent a first metatarsophalangeal joint arthrodesis by an IOFix implant for hallux rigidus

No	Age range	Year of surgery	Follow up (months)	Side	Time to union (months)	AOFAS score	Complications
1	56	2016	36	L	5	77	Scar anaesthesia
2	56	2016	36	L	8	83	Mild plantar flexion position
3	69	2016	37	L		85	
4	77	2016	40	R	4	87	
5	60	2015	48	R		67	A mild local discomfort, but no pain or signs of non-union
6	68	2015	48	L	9	35	Non-union
7	67	2015	46	L	8	80	
8	58	2015	45	R	7	83	
9	70	2018	14	R	6	90	
10	36	2015	54	L	20	87	Used Melmac for 8 months postoperatively
11	56	2017	24	R	–	83	Non-union
12	68	2015	52	L	12	80	
13	51	2015	42	R	6	87	
14	51	2015	40	L	9	75	
15	60	2015	38	R	6	83	
16	40	2017	25	L	9	87	Mild wound dehiscence
17	70	2018	14	L	8	69	A K-wire was inserted during the primary operation in order to add stability
18	56	2016	43	L	9	87	
19	58	2017	28	R	8	75	
20	58	2015	42	R	–	87	Non-symptomatic non-union
21	68	2016	38	R	10	87	
22	67	2016	39	L	8	87	
23	55	2015	53	L	6	85	
24	70	2017	25	R	8	87	
25	60	2016	38	R	10	69	
26	69	2017	26	L	8	85	
27	58	2015	52	R	8	87	
28	63	2016	38	L	9	80	
29	48	2016	12	R	10	Lost to follow up	
30	68	2015	13	L	9	Lost to follow up	
Mean	60.36 ± 9.12 (range 36 to 77) years		36.2 ± 12.31 (range 12 to 54) months		8.46 ± 2.93 (range 6 to 20) months	80.5 ± 10.87 (range 35 to 90) points	

*R* Right, *L* Left

**Fig. 5:**
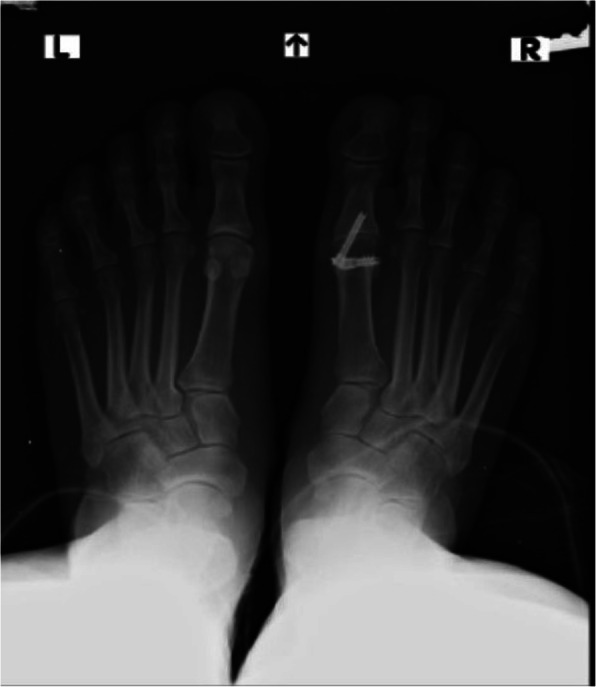
A non-symptomatic non-union of an arthrodesis of a first metatarso-phalangeal joint that was operated with an IOFix implant

Two patients had a non-union (Fig. 5). One was re-operated 8 months after his primary surgery due to a symptomatic nonunion that was resistant to a treatment with MELMAK™. We have performed a second fixation with plate and screws but union was still lacking. Eventually the patient was still mildly symptomatic and did not want a third surgery. The second patient presented with a non-symptomatic nonunion. Even at a 42 months postoperatively Plain radiograph have lacked any signs of fusion. Despite the radiographic appearance we did not detect painful movements in the involved joint, and no further surgery was indicated. Except for these two patients, all patients were satisfied with their results.

A more stringent approach would have been to regard the 2 patients who were treated with MELMAK™ as “failures”, and to exclude the 2 patients who were lost to follow up (even though a fusion was defined for both). This would have led to a 28 patient cohort and a fusion rate of 85.71% (24 patients).

## Discussion

In the current study we present a series of 30 patients who suffered from HR and underwent an MTPJ1 fusion with an IOFix device. Using this technique a very high fusion rate was achieved, although it wasn’t superior, and might have been even inferior to fusion rates with other implants. None of our patients had requested a hardware removal due to a prominent hardware during a minimum 2 years follow up. For our knowledge this is the largest series that was thus far published on HR patients who were operated by this implant. In a previous similar study on 21 patients by Singhal et al. [6], the use of IOFix allowed a 95% union rate. They too did not report on patients who requested an elective hardware removal. In a former preliminary series of 12 patients who were operated by an IOFix implant, the union rate was reported to be 91.67%. These two studies along with the current indicate reproducible union rates.

In the United states HR with arthritic changes was diagnosed in 2.5% of the population older than 50 years [1]. The pathophysiology of the disease is unclear. There are reports of familial history [8], but trauma [19], improper shoe wear [20], a tight Achilles tendon [20], or an elevated 1st metatarsus [8] were also proposed as possible triggers for the disease. In a series of 114 HR patients almost four of every five patients developed a bilateral disease, and 95% percent of the patients with a family history have eventually developed symptoms in the contralateral foot [8]. The most common presenting symptoms were found to be a local pain, and a decreased range of motion [8]. Physical examination often revealed a local tenderness, a dorsal bump, and a decreased range of motion [8]. A grind test exacerbated pain [8]. Standing radiographs can reveal degenerative changes that include metatarsal head flattening and widening, subchondral sclerosis, osteophytes and subchondral cysts, joint space narrowing, and eventually a joint destruction [8].

On the basis of the aforementioned clinical and radiographical findings Coughlin and Shurnas [8] have published a grading system according to which a treatment scheme was proposed. In their protocol non-surgical treatment was offered only for those patients who did not have any radiographical findings (grade 0). We on the contrary offered a primary non-surgical treatment to all of our patients as the first line of treatment for a period of 3 months. This treatment included Local anti-inflammatory drugs, activity modification (avoidance of tip toe standing, and avoidance of flexible sole shoes usage), and insoles with a Morton extension. The data on the patients who had radiographic signs of HR and were satisfied with a non-surgical treatment is beyond the scope of this article. Coughlin and Shurnas offered a cheilectomy procedure to all grade 1–3 patients, and added an arthrodesis as an option for grade 3 patients. Grade 4 patients were treated by an arthrodesis. We included grades 1–3 into the same “Moderate” group and have made the decision of whether to conduct a cheilectomy or an arthrodesis during operation according to the cartilage appearance. This was similar to the way Coughlin and Shurnas treated grade 3 patients. Severe cases, which were equivalent to Coughlin and Shurnas grade 4 were treated by an arthrodesis procedure [5] using an IOFix implant. We found this simplification of their grading system to be more applicable to the daily practice and similarly useful when treatment decisions were done.

A few modalities have been used in order to fuse the MTPJ1. With the use of plate and screws (cost~ 800 to 1200 Euro) in 233 ft, surgeons have obtained union in 230 (98%) joints [9]. Three (1.3%) of these 230 patients have suffered from a prominent hardware and underwent a secondary surgery for hardware removal. 26 (11.3%) patients have had minor complications (superficial wound infection, hematoma/seroma or mild wound dehiscence). With the use of staples (cost~ 600 Euro per 1 staple. At least two are usually needed), union was achieved in 29 (96.7%) out of 30 ft. [14] None of the 29 implants was removed. When two parallel screws (cost~ 200 Euro per 1 headless screw) were used in 60 ft a union rate of 100% was achieved and no hardware removal was reported [10]. Two crossed screws have led to a 93.3% union rate [12]. When only one intramedullary screw was used in 109 ft, union was achieved in 104 (95.4%) joints, but 85 (78%) of these patients have undergone a hardware removal due to a prominent hardware. This removal rate was exceptional, and alarmingly high. The last article was later criticized both for the questionable surgical technique and the authors’ determination for “fusion” [13]. Altogether, it seems that all fixation devices allowed similar fusion rates [13], and despite one report the removal rate was very low. In the authors hospital all mentioned surgeries are conducted in an elective outpatient setting. Patients arrive to the hospital during the morning hours, undergo the surgery under local anesthesia and are discharged on the same day. The staff and amenities, as well as the time needed to conduct these procedures are also similar. Accordingly, the implant is the only variable that can change the surgical cost. Since the IOFix (cost~ 2000 Euro) was more expensive, and did not allow superior results, its cost-effectiveness is questionable.

Biomechanically, the IOFix device was previously found to be superior over the crossed screws or a dorsal locking plate device when each of the three modalities was used as a single form of fixation [11]. In vitro the IOFix was shown to sustain higher loads, to be stiffer and to sustain a more narrow inter-fragmentary space [11]. In another biomechanical study [16] the IOFix implant, used as a single modality of fixation, was compared to a plantar or medial locking plate that were combined with a crossed screw. The IOFix was found to be superior in producing primary inter-fragmentary compression forces but inferior in failure susceptibility when compared to other fixation methods in that study.

The main weakness of this study is its retrospective nature and being non-comparative. We did not have a second treatment arm to compare the IOFix implant to, and could not therefore form a statistical model to assess our results, except for descriptive statistics. Although we presented postoperative AOFAS scores we were limited in evaluating the increase in this score values following operation since preoperative AOFAS scores were not available. Two patients were lost to follow-up. Although this is the largest series of its kind thus far, it is still a comparatively small patient group. Therefore, we might have had too few patients than the “number needed to harm”, when the need for reoperation was studied. A future study is mandated, which would be constructed as a prospective multiple-arm study that would compare clinical outcomes, and radiographical features such as union and alignment.

## Conclusions

The rates of fusion and hardware removal in MTPJ1 arthrodesis performed with an IOFix implant were found to be similar at most when compared to previously described fusion rates that were obtained with other cheaper and simpler fixation devices.

## Data Availability

The datasets used and/or analyzed during the current study are available from the corresponding author, David Segal MD, on reasonable request. The corresponding author can be reached at dudisegal@gmail.com

## Abbreviations

HR: Hallux rigidus
MTPJ1: First metatarsophalangeal joint
AOFAS: American orthopedic foot and ankle society
